# Delayed Poisoning by Anesthetics

**Published:** 1907-09

**Authors:** 


### DELAYED POISONING BY ANESTHETICS.

Guthrie summarizes his paper as follows: Operations should be delayed when possible if a fatty liver be suspected. It may be suspected in subjects of rickets and infantile paralysis who have been overfed with fattening food and under-exercised; in cases of sepsis and diabetes, and when a history of cylic vomiting is obtained. When a child has recently vomited apparently without cause, an intended operation should be postponed. This precaution has been neglected in many fatal cases recorded. When fatty liver is suspected the patient should be kept for some days on a diet restricted in fats. Bicarbonate or citrate of soda should be given meanwhile in order to neutralize fatty acids which may be present. Mild purgation may be beneficial. Starvation will give rise to acute acetonuria, therefore nutrient enemata should be given two hours before and immediately after an operation. Although any general anesthetic may be dangerous in the presence of a fatty liver, chloroform is most dangerous of all on account of its specific action on the liver and kidneys. The treatment of acid intoxication following operations should be by venesection, saline transfusion, and by clysters of solution of bicarbonate of sodium.—Clinical Journal, London, June 12th, 1907.

